# Relationship between subcellular localisation of Foscan® and caspase activation in photosensitised MCF-7 cells

**DOI:** 10.1038/sj.bjc.6603631

**Published:** 2007-02-27

**Authors:** S Marchal, A François, D Dumas, F Guillemin, L Bezdetnaya

**Affiliations:** 1 CRAN, Nancy University, CNRS UMR 7039, Centre Alexis Vautrin, Avenue de Bourgogne, 54511 Vandoeuvre-les-Nancy, France; 2 Faculté de Médecine, LEMTA, Nancy University, IFR 111 and CNRS UMR 7563, BP 184, 54505 Vandœuvre-les-Nancy, France

**Keywords:** photodynamic therapy, Foscan®, subcellular localisation, endoplasmic reticulum stress, apoptosis, caspase-7

## Abstract

The present study investigates the relationship between the subcellular localisation of Foscan® and intrinsic apoptotic pathway post Foscan®-based photodynamic therapy (PDT). With this purpose, mammary carcinoma MCF-7 cells were incubated with Foscan® for 3 or 24 h and then subjected to equitoxic light doses. Fluorescence microscopy revealed very good Foscan® co-localization to endoplasmic reticulum (ER) and Golgi apparatus after 3 h incubation with MCF-7 cells. Progressive increase in incubation time shows leakage of Foscan® from Golgi apparatus. Twenty-four hours incubation yielded a fluence-dependent enhanced induction of the ER-resident glucose-regulated protein 78 (Bip/GRP78), along with a weak mitochondrial damage, thus underscoring the ER as the main site of photodamage after prolonged incubation. Analysis of events implicated in apoptotic pathway after 24 h incubation demonstrated photodamage to Bcl-2 protein in total cellular extract, but not in the mitochondrial fraction. We further determined an increase in caspases-7 and -6 activation, which was strongly related to the expression of GRP78. The above findings demonstrate that Foscan® localisation in ER improves the photoactivation of the caspase-7 apoptotic pathway, which is poorly related to mitochondrial damage.

Photodynamic therapy (PDT) has been approved as a treatment modality in various cancerous and non-cancerous lesions (Dougherty, 2002). Photodynamic therapy, through the combination of three elements, a photosensitising agent, light and oxygen, triggers the liberation of highly reactive oxygen species, leading to damage of cellular components and eventually to apoptotic and/or necrotic cell death.

Several distinct apoptotic pathways induced by photooxidative stress have recently been established (Oleinick *et al*, 2002). These include the death-receptor activation at the surface of tumour cells (extrinsic pathway) or mitochondrial outer membrane permeabilisation (intrinsic pathway). Intrinsic apoptosis can be initiated from signals that originate from or converge to intracellular organelles. An important role in the initiation of intrinsic apoptosis following PDT has been attributed to oxidative stress of the endoplasmic reticulum (ER), which proceeds through disruption of calcium homeostasis and accumulation of misfolded proteins (Mak *et al*, 2004; Wong *et al*, 2004; Kessel *et al*, 2005; Buytaert *et al*, 2006). How and to what extent photodamaged-ER contributes to apoptosis induced by PDT is still under investigation.

The short migration distance of singlet oxygen (less than 0.02 *μ*m) suggests a narrow relationship between the sites of subcellular localisation of the photosensitiser and photodamage to nearby organelles involved in apoptotic and/or necrotic cell death (Oleinick *et al*, 2002). It has been assumed that photosensitisers primarily localised in mitochondria are able to induce early apoptosis by rapid loss of mitochondrial transmembrane potential and/or release of apoptosis-inducing factors such as cytochrome c (cyt *c*), itself leading to post-mitochondrial caspase activation (Kessel and Luo, 1999; Chiu and Oleinick, 2001). Several reports investigated the possibility to modulate the mechanism of PDT-induced cell death using protocols that selectively target proapoptotic organelles (Fabris *et al*, 2001; Hsieh *et al*, 2003). Prolonged incubation of photosensitisers (24 h) with cells compared with shorter incubation times (2–3 h) was accompanied by a more efficient apoptosis after photoirradiation. This was attributed to photosensitiser relocalisation to mitochondria or Golgi apparatus during incubation (Fabris *et al*, 2001; Hsieh *et al*, 2003).

Foscan® (meta-tetrahydroxyphenylchlorin), a second-generation photosensitiser has been granted European approval for palliative treatment of advanced head and neck cancers. The efficacy of Foscan®–PDT in the treatment of early squamous cell carcinoma (Hopper *et al*, 2004; Lou *et al*, 2004) and other malignancies has also been recently reported (Campbell *et al*, 2004; Lovat *et al*, 2005; Shikowitz *et al*, 2005). Endoplasmic reticulum and Golgi apparatus have been demonstrated as preferential sites of Foscan® accumulation in cultured tumour cells after 3-h incubation (Teiten *et al*, 2003a) leading to primary photodamage of these organelles upon irradiation (Teiten *et al*, 2003b). Despite that mitochondria are not specifically targeted by Foscan®, a fluence-dependent mitochondrial depolarisation has been observed, concomitant with cyt *c* release immediately after Foscan®-based PDT (Chen *et al*, 2000; Teiten *et al*, 2003b; Marchal *et al*, 2005) and consecutive post-mitochondrial apoptotic induction (Chen *et al*, 2000; Marchal *et al*, 2005). These results have suggested that initiating apoptotic events originate from ER/Golgi and/or mitochondria. The present study investigates the relationship between the subcellular localisation of Foscan® and intrinsic apoptotic pathway post Foscan®-based PDT. With this purpose, we specified subcellular distribution of Foscan® with time in mammary carcinoma MCF-7 cells and evaluated photoinduced caspases activation in relation to the dye subcellular localisation.

## MATERIALS AND METHODS

### Chemicals

Foscan® was provided by Biolitec AG (Jena, Germany). Specific organelle fluorescent probes DiOC_6_ (3) (3,3′-dihexyloxacarbocyanine iodide), BODIPY FL C_5_ ceramide (BPC), MitoTracker Green and the green fluorescent probe 5,5′,6,6′-tetrachloro-1,1′,3,3′ tetraethylbenzimidazolylcarbocyanine iodide (JC-1) were purchased from Invitrogen Molecular Probes, Cergy-Pontoise, France. APO2.7 phycoerythrin (PE)-conjugated monoclonal antibody (Beckman Coulter, Roissy, France) and the 5,5′,6,6′-tetrachloro-1,1′,3,3′-tetraethylbenzimidazolyl-carbocyanine iodide (JC-1) (Invitrogen Molecular Probes) were used to monitor mitochondrial dysfunction. Rabbit anti-cleaved caspase-7, rabbit anti-cleaved caspase-6, rabbit anti-cleaved caspase-9 antibodies and horseradish peroxidase (HRP)-conjugated anti-mouse or rabbit IgG antibody were obtained from Cell Signaling, Danvers, MA, USA. Mouse anti-Bcl-2 mouse, anti-cleaved poly-ADP-ribose polymerase (PARP), mouse anti-Bip/GRP78 (glucose-regulated protein 78) antibodies, caspase-positive control (camptothecin-treated Jurkat cells) and Ac-DEVD-CHO caspase-3/-7 inhibitor were purchased from BD Biosciences, Le-Pont-de-Claix, France. Goat anti-actin was provided by Tebu-bio, Le-Perray-en-Yvelines, France.

### Cells

The human breast adenocarcinoma cell line MCF-7 was grown in phenol red-free RPMI 1640 medium (Invitrogen, Cergy-Pontoise, France) supplemented with 9% (v v^−1^) heat-inactivated fetal calf serum (FCS) (PAN Biotech GmbH, Aidenbach, Germany), 1% (v v^−1^) penicillin (10 000 IU) streptomycin (10 000 *μ*g ml^−1^) and 1% (v v^−1^) glutamin 200 × 10^−3^ M (Invitrogen). Cells were kept as monolayer culture in a humidified incubator (5% (v v^−1^) CO_2_ in air) at 37°C. Cell cultures were re-seeded every week to ensure an exponential growth.

### Photosensitiser

Foscan® stock solution (6 × 10^−3^ M) was performed in a solvent mixture of ethanol/polyethylene glycol/water (20 : 30 : 50, by vol) and was kept at 4°C in the dark. Further dilution was performed in RPMI 1640 medium supplemented with 2% (v v^−1^) FCS to reach a final Foscan® concentration of 1.5 × 10^−6^ M.

### Confocal laser scanning microscopy

MCF-7 cells (1 × 10^4^ cells ml^−1^) were plated into eight-well chambers Slideflask (Nunc, Roskilde, Denmark), incubated in the dark at 37°C with 1.5 × 10^−6^ M Foscan® for different times (from 3 to 24 h), then rinsed in the medium and incubated with organelle-specific fluorescent probes. The ER was labelled with DiOC_6_ dye, applied for 1 min at a final concentration of 2.5 *μ*g ml^−1^. To identify Golgi apparatus, cells were labelled with 5 × 10^−6^ M BPC for 30 min at 4°C, then rinsed with Hank's buffered saline solution containing 10 × 10^−3^ M *N*-2-hydroxyethylpiperazine-*N*′-2-ethanesulphonic acid (HEPES), pH 7.4 (Hanks’ balanced salt solution (HBSS)/HEPES) and re-incubated at 37°C for the next 30 min. The staining of mitochondria was performed by cells incubation with 500 × 10^−9^ M MitoTracker Green for 30 min at 37°C. Before observation, dyes were removed by gentle rinsing with RPMI 1640 or HBSS/HEPES buffer for BPC. Double-stained cells were observed with a confocal laser-scanning microscope (SP2 AOBS LCSM, Leica microsystem, Wetzlar, Germany). An optimal pinhole size of 60.64 *μ*m was used to exclude fluorescence light emitted from out-of-focus plane above and below the focusing plane. An oil immersion objective (× 63) was used to capture each image of 512 × 512 pixels size. Confocal microscopy was coupled with microspectrofluorimetry to define spectral profiles of Foscan® and organelle probes in the same focal plane. Organelle-specific fluorescent probes were excited with a 488 nm argon/argon krypton laser and Foscan® was excited with a helium/neon laser at 633 nm. Band-pass emission filters of 505–550 and 640–660 nm were respectively used to discriminate organelle probes (channel 1, green) from Foscan® (channel 2, red) fluorescence.

### Measurements of Foscan® intracellular concentrations

MCF-7 cells were incubated with 1.5 × 10^−6^ M Foscan® for 3 or 24 h. After incubation, cells were washed twice with cold phosphate-buffered saline (PBS), trypsinised, washed in PBS, resuspended in methanol and sonicated for 10 min. After centrifugation (3500 r.p.m., 5 min), supernatant was introduced into a 10 × 10-mm quartz cuvette. Fluorescence intensity from cells lysates was measured with respect to a calibration curve ranging from 0 to 0.45 × 10^−6^ M Foscan® in methanol. Fluorescence intensity of each sample was normalised to the protein content determined by the DC protein assay (Bio-Rad laboratories, Marnes-la-Coquette, France). Emission spectra were carried out using SAFAS luminescence spectrometer. Spectra were collected between 600 and 700 nm (excitation wavelength 422 nm; photomultiplier voltage 650 V).

Foscan® mitochondria concentration was assessed according to Laville *et al* (2003). Briefly, mitochondria were isolated by differential centrifugation in 0.25 M ice-cold sucrose solution. Cell membranes were disrupted, centrifuged and the supernatant was further centrifuged for an additional 15 min at 6800 *g*. Sucrose was added to the pellet and the suspension was centrifuged twice for 15 min at 10 000 *g*. The last pellet consisted of mitochondrial fraction. Foscan® concentration was measured by spectrofluorimetry according the procedure described above.

Mitochondrial preparations were analysed for contamination with ER by Western blotting for ER-marker GRP 78. By such analysis, mitochondrial fraction was judged to be >75% free of ER contamination.

### Photodynamic treatment

Four days before treatment, 4 × 10^4^ cells ml^−1^ were seeded in Petri dishes and then logarithmically growing MCF-7 cells were washed twice and incubated with fresh medium containing 2% (v v^−1^) FCS with 1.5 × 10^−6^ M Foscan® for 3 or 24 h before light exposure. Before photosensitisation, cells were washed three times, incubated with RPMI 9% (v v^−1^) FCS, then irradiated at room temperature with a 650-nm laser diode (F-System, Coherent) at a fixed fluence rate of 2.12 mW cm^−2^. Following irradiation, cells were maintained in a humidified 5% (v v^−1^) CO_2_ incubator at 37°C for various times periods until experiments.

### Cell viability assay

Cell viability was assessed by the clonogenic assay. Logarithmically growing MCF-7 cells were trypsinised immediately after PDT, seeded in triplicate into six-well plates at a density of 500 cells per well. Nine days after treatment, medium was removed, colonies were fixed with 70% (v v^−1^) ethanol and stained with 1% (w v^−1^) crystal violet (Pointet Girard, Clichy, France) for 5 min. Dye excess was carefully washed off and colonies composed of more than 50 cells were counted with a robotised image analysis system (Clemex, Longueil, Canada). Each experiment was done at least three times. Cell death percentage was obtained by referring treated samples to non-irradiated culture (drug, no light).

### Immunoblotting analysis

For immunoblotting analysis, unless otherwise indicated, MCF-7 cells were collected by scraping immediately, 4 and 24 h after Foscan®–PDT. When experiments were performed in the presence of caspase-3/7 inhibitor, a 20 × 10^−6^ M Ac-DEVD-CHO was added to cells 30 min before irradiation and maintained with cells until analysis.

The procedure used for the Western blot detection has been described in detail (Marchal *et al*, 2005). Briefly, after protein extraction, the samples were subjected to electrophoresis in SDS-polyacrylamide gels (SDS-PAGE gels). Before immunoblotting, nonspecific binding was blocked with 0.1% (v v^−1^) Tween-20 in Tris-buffered saline containing 5% (wv^−1^) non-fat dry milk for 1 h at room temperature. Afterwards, membranes were probed overnight at 4°C with an adapted concentration of each antibody followed by the appropriate HRP-conjugated antibody for 1 h at room temperature (1 : 2000). The immune complexes were detected by enhanced chemiluminescence system (GE Healthcare, Orsay, France) and visualised by autoradiography.

Probing with a mouse anti-actin antibody was used as loading control for the blots.

### Flow cytometry analysis of cyt c release and mitochondrial membrane depolarization

The measurements of cyt *c* release and mitochondrial membrane depolarization (Δ*φ*_m_) after PDT was assessed by flow cytometry technique (FACS Calibur, BD BioSciences) as described previously (Teiten *et al*, 2003b). Briefly, for measurements of cyt *c* release, permeabilised cells were labelled with PE-APO2.7 for 15 min at room temperature and subjected to flow cytometry analysis (*λ*_ex_=488; *λ*_em_=585±42 nm; FL2).

The measurement of Δ*φ*_m_ was performed by the use of JC-1 probe. Cells were centrifuged, the cell pellet was suspended in 1 ml medium containing 1 *μ*l of JC-1 (final concentration 5 *μ*g ml^−1^) and the resulting suspension was measured by flow cytometry after a 15-min incubation at 37°C. The fluorescence of JC-1 mitochondria-sequestered aggregates (*λ*_ex/em_=488/590 nm) was detected in channel FL2 with a 585±42-nm band–pass, whereas cytoplasmic monomer fluorescence (*λ*_ex/em_=488/527 nm) was detected in channel FL1 with a 530±30-nm band-pass filter.

### Statistical analysis

Mann–Whitney's *U*-test was employed to determine the statistical significance with a limit set to *P*<0.05 using Staview 5.0 software.

## RESULTS

### Foscan® subcellular localisation in respect to time of incubation

**Figure 1:**
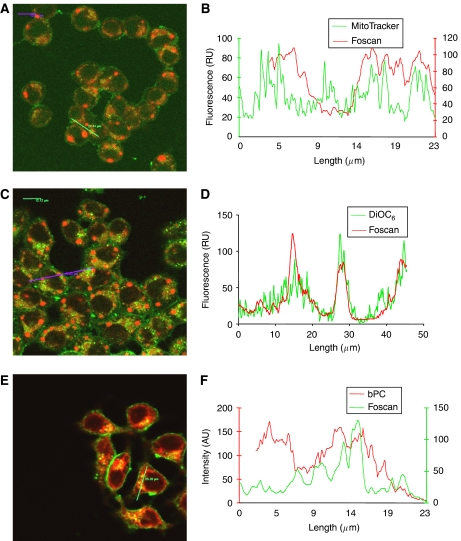
Foscan® localisation in MCF-7 cells. (**A**, **C**, **E**; left panel) Confocal overlay images and (**B**, **D**, **F**; right panel) fluorescence topographic profiles of MCF-7 cells double-stained with Foscan® and organelle probes. Arrow indicates the analysed longitudinal transcellular zone. Cells were sensitised with 1.5 × 10^−6^ M Foscani® for 24 h, washed and subjected to organelle staining. (**A**, **B**) Mitochondria were labeled with 500 × 10^−9^ M MitoTracker green for 30 min at 37°C. (**C**, **D**) ER was stained with 2.5 *μ*g ml^−1^ DiOC6 for 1 min. (**E**, **F**) Golgi apparatus was labeled with 5 × 10^−6^ M BPC for 30 min at 4°C. Organelle-specific probes were excited with a 488-nm; Foscan® was excited at 633 nm. Objective magnification × 63.

In our previous study, we demonstrated that after 3 h incubation, Foscan localised primarily in the Golgi apparatus and ER of MCF-7 cells (Teiten *et al*, 2003a). In the present study, Foscan localisation in MCF-7 cells after 24 h incubation, assessed by confocal images of double-stained cells with Foscan® and specific-organelle fluorescent probes together with topographic profiles are depicted in Figure 1. Good superposition between Foscan® and specific probes is revealed in yellow.

**Figure 2:**
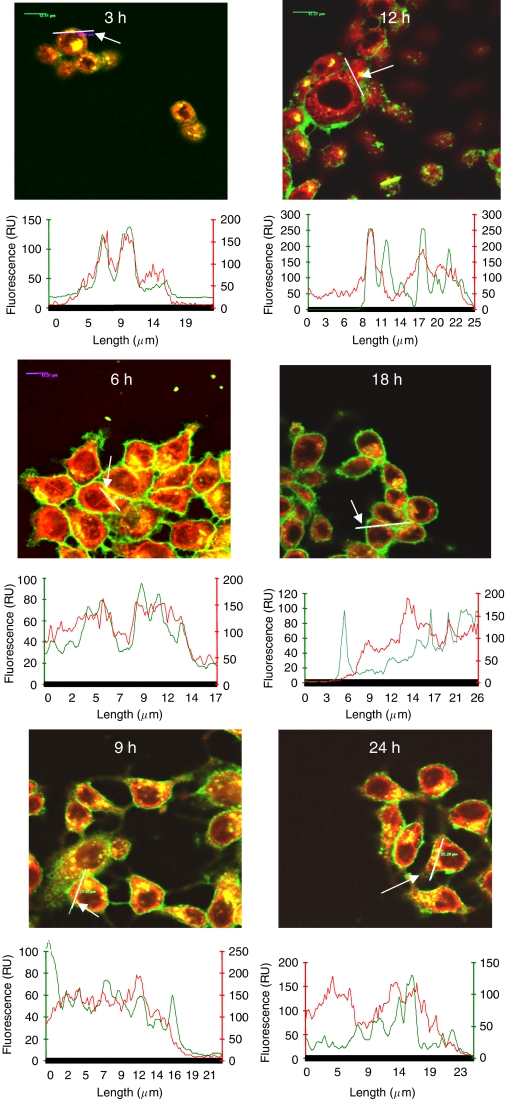
Kinetic of Foscan® localisation in Golgi apparatus in MCF-7 cells. Cells were sensitised with 1.5 × 10^−6^ M Foscan® from 3 to 24 h incubation time, washed and subjected to staining with 5 × 10^−6^ M BPC for 30 min at 4°C. Fluorescence topographic profiles of MCF-7 cells double-stained with Foscan® and BPC are presented under confocal overlay images of MCF-7 cells double-stained with BPC (arrow indicates the analysed longitudinal transcellular zone). Organelle-specific probes were excited at 488 nm; Foscan® was excited at 633 nm. Objective magnification × 63.

Co-staining images and topographic profiles of Foscan® and MitoTracker green revealed a weak correlation, thus pointing out a scarce localisation of Foscan® in mitochondria (Figure 1A and B). At the same time, Foscan® was particularly well localised in ER, as demonstrated by the perfect overlap of Foscan® and DiOC6 in topographic profiles (Figure 1D). In contrast to 3 h incubation, characterised by the good dye localisation in Golgi apparatus (Teiten *et al*, 2003a), the staining patterns and topographic profiles of Golgi probe BPC and Foscan after 24 h incubation did not show any overlap (Figure 1E and F). Foscan® accumulation in the Golgi apparatus was further mapped in the time span of 3–24 h (Figure 2). After 3–6 h incubation, a good localisation of Foscan® in the Golgi apparatus was evidenced by a yellow pattern in the dual-staining images and a very good overlap in fluorescence topographic profiles (Figure 2). However, from 12 h incubation a progressive extrusion of Foscan® from the Golgi becomes noticeable with increasing mismatch between Foscan® and BPC images and profiles.

### Fluence-dependent cell photoinactivation with respect to incubation time

Cells incubated with Foscan® for 3–24 h were subjected to the range of light fluences and their photocytotoxicity was further assessed by clonogenic assay. Table 1 displays the light fluences at which the same levels of photocytotoxicity (equitoxic light doses) were achieved under both incubation conditions. For all selected lethal doses (from LD_63_ to LD_97_), an incubation time of 24 h necessitated a six- to 12-fold lower light fluence than 3 h incubation.

### GRP78 protein photoinduction in MCF-7 cells with respect to incubation time

**Figure 3:**
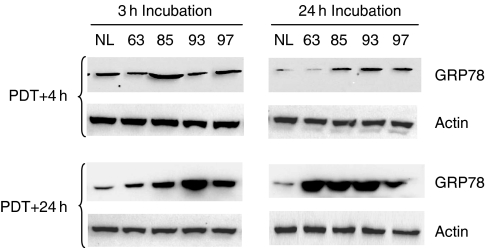
Fluence-dependent GRP78 protein expression in MCF-7 cells. (**A**) MCF-7 cells were incubated with 1.5 × 10^−6^ M Foscan® for 3 h or (**B**) 24 h and subjected to equitoxic light doses. Western Blotting of GRP78 and actin protein expression was determined from cell lysates at 4 and 24 h after PDT. Control cells (NL) were subjected to Foscan® only (drug, no light).

GRP78 induction, a stress associated ER resident protein, was assessed 4 h and 24 h post-PDT after both incubation times using Western blotting (Figure 3). Cells subjected to short incubation periods with Foscan® did not reveal any changes in GRP78 expression 4 h post-PDT (Figure 3A), whereas an upregulation of GRP78 was observed at all light fluences 24 h after illumination (Figure 3A). When cells were incubated with the dye for 24 h, protein induction occurred as soon as 4 h post-PDT starting from LD_85_ and was significantly enhanced at all light doses 24 h post-PDT (Figure 3B).

### Mitochondria and total Foscan® intracellular content

Table 2 presents Foscan® content measured by spectrofluorimetry following extraction from whole cells and mitochondria. After 3 and 24 h incubation, whole cellular content was 9.4±0.4 × 10^−11^ mol Foscan® mg^−1^ protein and 76.0±5.7 × 10^−11^ mol Foscan® mg^−1^ protein, respectively. Foscan® concentrations in mitochondria were 3.3±2.2 × 10^−11^ mol Foscan® mg^−1^ protein and 7.7±2.7 × 10^−11^ mol Foscan® mg^−1^ protein, respectively, for 3 and 24 h incubation. The mitochondrial fraction of Foscan® represented 33.6±3.7 and 10.2±1.2% of the total cellular content at 3 and 24 h incubation, respectively.

### Photoinduced mitochondrial damage with respect to incubation time

Flow cytometry evaluation of cyt *c* release and collapse of *Δφ*_m_ from photodynamically treated cells were performed immediately 24 h post-PDT.

**Figure 4:**
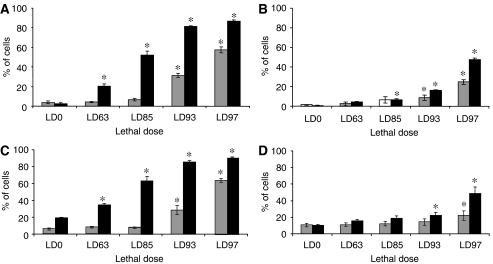
Fluence-dependent mitochondria membrane photodamage. (**A**, **B**) Fluence-dependent cyt *c* release and (**C**, **D**) mitochondrial membrane depolarisation in Foscan®-photosensitised MCF-7 cells. MCF-7 cells were incubated with 1.5 × 10^−6^ M Foscan® for (**A**, **C**) 3 h or (**B**, **D**) 24 h and subjected to equitoxic light doses. Photosensitised MCF-7 cells were analysed immediately (□) and 24 h after irradiation (▪). Results are the mean±s.e.m. of at least three independent experiments. ^*^, significantly different from control values.

Mitochondrial damage in cells incubated 3 h with Foscan® and assessed immediately after PDT was dose-dependent as shown in Figure 4A and C. For low fluences (LD_63_ and LD_85_) both cyt *c* release and Δ*φ*_m_ was not significantly different from control cells (*P*>0.05), whereas at LD_93_ and LD_97_ we observed a significant increase in both parameters (Figure 4A and C). Mitochondrial damage 24 h post-PDT demonstrated a significant dose-dependent increase in cyt *c* release and loss of *Δφ*_m_ (Figure 4A and C).

Cells subjected to 24 h incubation with Foscan® demonstrated much less pronounced mitochondrial photodamage compared with 3 h incubation, irrespective of the time post-PDT (Figure 4B and D). For instance, measurements carried out 24 h post-PDT showed from 5 (LD_63_) to 45% (LD_97_) of damaged cell (Figure 4B and D), whereas equitoxic doses applied after 3 h incubation resulted in mitochondrial damage of 20 and 85% of cells (Figure 4A and C).

Therefore, the mitochondrial response was considerably decreased when cells were submitted to prolonged Foscan® incubation.

### Activation of post-mitochondrial caspases cascade pathway

We further investigated the activation of post-mitochondrial apoptotic events by Western blot analysis of the proteolytic cleavage of caspases -9, -7, -6 and PARP in Foscan® photosensitised cells at 24 h post-PDT.

**Figure 5:**
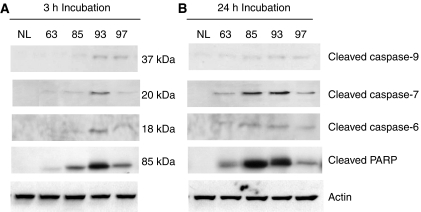
Fluence-dependent caspases activation in Foscan®-photosensitised MCF-7 cells. (**A**) MCF-7 cells were incubated with 1.5 × 10^−6^ M Foscan® for 3 h or (**B**) 24 h and subjected to equitoxic light doses. Western blotting was determined from cell lysates at 24 h after PDT by using specific antibodies of caspase-9, -7, -6 and PARP-cleaved forms. Control cells (NL) were subjected to Foscan® only (drug, no light).

Immunoblotting revealed a dose-dependent cleavage of caspases-9, -7, -6 and PARP after both incubation times (Figure 5) with an increased expression at first three light fluences, followed by reduced cleavage at the highest applied fluence. Compared with cleaved caspase-7 and PARP, caspase-9- and caspase-6-cleaved fragments displayed discrete expression. For long incubation times, expression of cleaved caspase-7, -6 and PARP was obviously higher and enzyme cleavage occurred at lower light doses (LD_63_ for 24 h *vs* LD_93_ for 3 h).

### Effect of Foscan®–PDT on Bcl-2 protein expression

**Figure 6:**
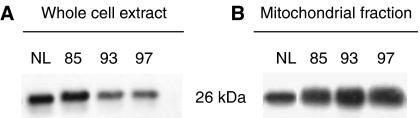
Fluence-dependent Bcl-2 expression in Foscan®-photosensitised MCF-7 cells. (**A**) From whole cell extract and (**B**) from mitochondrial fraction. Before irradiation, MCF-7 cells were incubated with 1.5 × 10^−6^ M Foscan® for 24 h. Western blotting was determined from cell lysates immediately after PDT. Control cells (NL) were subjected to Foscan® only (drug, no light).

Expression of Bcl-2 protein was assessed immediately after irradiation of cells preloaded with Foscan® for 24 h as well as from whole cell extract from mitochondrial fraction (Figure 6). Bcl-2 measured in the whole extract was unaffected at LD_85_ but decreased with increasing light doses. In opposition, this protein remained unchanged at all applied fluences in mitochondrial fraction.

## DISCUSSION

Mechanism of cell death induced by photooxidative stress is tightly related to the sites of intracellular photosensitiser accumulation. As such, a better comprehension of the role of specific organelles in mediating apoptotic photoinduced response provides a possibility to manipulate the cell death machinery.

Confocal microscopy investigation of Foscan® intracellular distribution together with the evaluation of enzymatic post-irradiation activity of selected organelles after 3 h incubation provided an unambiguous evidence of Foscan® localisation in the ER and Golgi apparatus (Teiten *et al*, 2003a). In the present report, confocal microscopy investigation of Foscan® intracellular distribution showed that prolonging incubation time to 24 h induced variations in its subcellular distribution. Very good Foscan® localisation in ER along with a weak distribution in mitochondria was maintained with time (Figure 1). The main difference observed was the poor localisation in the Golgi apparatus after 24 h, suggesting extrusion of Foscan® from Golgi with time. Cogent evidence was given by kinetic of Foscan® relocalisation over a 24-h period. Good localisation of Foscan® in Golgi apparatus observed at 3 h incubation vanished progressively with time and starting from 12 h, the BPC and Foscan® fluorescence profiles were clearly different, indicating a poor affinity of Foscan® for this organelle (Figure 2). Based on this localisation pattern, and also that only 10% of total intracellular Foscan® content was found in mitochondria (Table 2), we supposed a predominant accumulation of Foscan® in the ER after 24 h incubation. Further confirmation of the enhanced presence of Foscan® in the ER was obtained in the study of the ER-resident GRP78 protein expression after PDT. This chaperone protein is known to be induced by the unfolded protein response to alleviate ER stress and maintain cell survival (Rao *et al*, 2002). By using mitochondria- and/or ER-targeting photosensitisers, dose-dependent elevation of GRP78 has been observed in few hours post-irradiation, thus assuming a classic ER oxidative stress response following PDT (Mak *et al*, 2004; Wong *et al*, 2004). Compared with cells incubated for 3 h, cells incubated with Foscan® for 24 h and subjected to equitoxic light doses exhibited higher upregulation of GRP78 (Figure 3). These combined results indicate that prolonged incubation favours ER localisation. It is pertinent to notice that clinical therapy with Foscan® uses 96 h time interval between administration and irradiation suggesting that the ultimate localisation pattern involves the ER.

Short incubation time favoured massive mitochondrial membrane injury irrespective of time post-PDT (Figure 4). Moreover, all light fluences, except the lowest one (LD_63_), yielded significant cyt *c* release and collapse of *Δφ*_m_ right after PDT (Figure 4A and C), thus presuming direct mitochondrial photodamage. Alternative explanation for the cyt *c* release and loss of *Δφ*_m_ could be a translocation of the proapoptotic protein Bax during irradiation (Kessel and Castelli, 2001). The process of the Bax insertion into the mitochondrial membrane is sensitive to temperature (Pryde *et al*, 2000). Therefore, in the next step we conducted both cell irradiation (LD_93_) and measurements of *Δφ*_m_ at 15°C, the temperature at which Bax penetration into mitochondria is suppressed. Upon these conditions, we observed an immediate loss of *Δφ*_m_ comparable with that at 37°C (27.3±4.2 *vs* 33.3±6.1%), thus ruling out indirect mitochondria damage.

We studied further the apoptotic events induced by PDT in relation to specific Foscan® localisation.

Considering that MCF-7 cells are deficient in caspase-3, they are able to undergo apoptosis through pathways different from caspase-3 activation (Janicke *et al*, 1998). Caspases-6 and -7 may partially substitute caspase-3 in these cells suggesting that post-mitochondrial cascade of caspases activation, successively involving caspases-9, -7 and -6, governs apoptosis in MCF-7 cells (Slee *et al*, 2001).

It has been shown that PDT-induced activation of caspase-9 requires the cyt *c* release from mitochondria (Oleinick *et al*, 2002). The massive photoinduced cyt *c* release after 3 h, but not after 24 h incubation (Figure 4A), may suggest an elevated expression of cleaved caspase-9 under 3 h incubation. However, Figure 5 indicates a weak expression of cleaved caspase-9, without obvious difference in its activation at both incubation times. A plausible explanation could be an observation of the close relationship between the presence of active caspase-3 and procaspase-9 processing. Indeed, cyt *c*-mediated processing of procaspase-9 was reported to be strongly impaired in caspase-3-deficient cells (Blanc *et al*, 2000; Xue *et al*, 2001b).

Activation of caspases 6, -7 and PARP cleavage were studied 24 h post-PDT (Figure 5). For both incubation times we observed the cleavage of caspases 6, -7 and PARP at all light doses, except the highest one (Figure 5). This could be related to the inhibition of apoptosis in favour of necrosis on excess of oxidative damage (Marchal *et al*, 2005). In the presence of Ac-DEVD-CHO, a specific inhibitor of caspase-7, a complete inhibition of the proteolytic process of caspase-7, -6 and PARP was registered (data not shown), thus indicating that apoptotic pathway was primarily governed by caspase-7. The major role of caspase-7 in apoptotic mechanism in MCF-7 cells has been already reported (Mooney *et al*, 2002).

Compared to 3 h incubation, Western blotting demonstrated much stronger expression of cleaved caspases-6, -7 and PARP in cells subjected to equitoxic doses after 24 h incubation (Figure 5A and B). These results are consistent with the improved GRP78 induction (Figure 3) along with the weak mitochondrial damage (Figure 4B) in cells after prolonged incubation. The mechanism underlying ER stress-associated apoptosis is poorly understood. It has been proposed recently that caspase-7 could be involved in ER –stress-induced apoptosis through its association with a subpopulation of GRP78 existing as an ER transmembrane protein (Reddy *et al*, 2003; Rao *et al*, 2004; Wu *et al*, 2005). Basically, caspase-7 and GRP78 bind to each other (Wu *et al*, 2005) preventing the activation of caspase-7 (Reddy *et al*, 2003). However, under severe ER stress the complex could disrupt resulting in caspase-7 activation (Rao *et al*, 2002; Wu *et al*, 2005). This explanation can also stand for the results obtained in the present study and account for an apoptotic pathway that is different from the classical post-mitochondrial process. Another scenario for the observed apoptotic effects could be offered by the photodamage to Bcl-2 protein. Photochemical destruction of both mitochondrial- and ER-localised Bcl-2 followed by apoptosis has been demonstrated in several studies (Kessel and Castelli, 2001; Xue *et al*, 2001a). Therefore, rther examined Bcl-2 photodamage after prolonged incubation of cells with Foscan®. We observed dose-dependent loss of Bcl-2 in whole cell extract, whereas no obvious signs of its photodestruction were noticed in mitochondrial fraction (Figure 6). These results point out that ER-localised Bcl-2 could be a possible target of Foscan®-induced photodamage. It has recently been demonstrated that ER-resident Bcl-2 controls apoptosis through sequestration/inactivation of proapototic mitochondria BH3-only proteins that activate Bax (Thomenius *et al*, 2003). However, we failed to establish a clear relationship between Bcl-2 photodestruction and caspases activation as caspase-6, -7 and PARP-cleavage was maximum at LD_85_ (Figure 5B), whereas expression of Bcl-2 was unaffected at this fluence (Figure 6A).

The duration of photosensitiser contact with the tissue is a probable determining parameter in adjusting cell-death mechanism. Applying protocols that elicit ER as the main target of Foscan®-mediated PDT could be an efficient proapoptotic strategy.

## Figures and Tables

**Table 1:** Light fluences (J cm^−2^) and corresponding irradiation times (s) inducing the same level of photocytotoxicity in MCF-7 cells under 3 and 24 h incubation with Foscan®

	**Fluence, J cm^−2^ (irradiation time, s)**	
**Photocytotoxicity^a^^,^^b^**	**3 h**	**24 h**
63% (±17.1)	0.06 (30)	0.011 (5)
85% (±8.8)	0.13 (60)	0.021 (10)
93% (±6.3)	0.38 (180)	0.032 (20)
97% (±2.2)	0.74 (330)	0.064 (30)

a Photocytotoxicity of Foscan® was assessed *in vitro* using clonogenic assay. Cells were incubated with 1.5 × 10^−^6 M Foscan® for 3 or 24 h before irradiation with light doses producing 63 (LD_63_), 85 (LD_85_), 93 (LD_93_) and 97 (LD_97_) cell mortality.

b Mean±s.d. (in italic) of at least triplicate experiments.

**Table 2:** Foscan® concentration^a^^,^^b^ in whole cells and in mitochondria fraction with respect to the time of incubation with MCF-7 cells

	**3 h**	**24 h**
	**mol mg protein^−1^**	**mol mg protein^−1^**
Total cell concentration	9.4 × 10^−11^±0.4	76.0 × 10^−11^±5.7
Mitochondria concentration	3.3 × 10^−11^±2.2	7.7 × 10^−11^±2.7
Relative content^c^	33.6±3.7%^c^	10.2±1.2%^c^

a Foscan® concentration was assessed by fluorescence spectrometry from cell lysate.

b Mean±s.d. of at least triplicate determinations.

c Relative content calculated as the percentage of Foscan® concentration in mitochondria to the intracellular Foscan® concentration.
